# Identifying Candidate Genes Related to the Nutritional Components of Soybean (*Glycine max*) Sprouts Based on the Transcriptome and Co-Expression Network

**DOI:** 10.3390/genes16060692

**Published:** 2025-06-06

**Authors:** Cheng Wang, Qiaoli Hu, Yan Wang, Shulin Lan, Xueting Li, Hui Liu, Xue Feng, Qiaoxia Shang, Weiyu Li

**Affiliations:** 1 College of Plant Science and Technology, Beijing Key Laboratory of New Agricultural Technology in Agriculture Application, National Demonstration Center for Experimental Plant Production Education, Beijing University of Agriculture, Beijing 102206, China; wangcheng1983@163.com (C.W.); lixueting@bua.edu.cn (X.L.); 202220221092@bua.edu.cn (H.L.);; 2 Key Laboratory for Northern Urban Agriculture of Ministry of Agriculture and Rural Affairs, Beijing University of Agriculture, Beijing 102206, China; w1427783723@126.com

**Keywords:** soybean, bean sprouts, transcriptome, WGCNA, candidate genes

## Abstract

Background: During the germination of soybean seeds, many biochemical metabolic reactions become extremely active, resulting in a series of physiological and biochemical activities, and the seeds being rich in nutrients. Studying the network and key genes that regulate the nutritional content of bean sprouts is particularly important. Methods: In this study, the nutrient contents of Dongnong 254 and Heze small beans were measured when the bean sprouts were 1 cm, 3 cm, 5 cm and 7 cm long, and transcriptome sequencing was performed. Results: Clustering and principal component analysis (PCA) revealed that the samples could be divided into three groups. The differences between Dongnong 254 and Heze small bean samples with sprout lengths of 5 cm and 7 cm were greater than those between materials. Through differential expression analysis, 18,472 differentially expressed genes (DEGs) in the material included 1816 unique DEGs, and a total of six clusters with statistical significance were identified, which were enriched in pathways related to photosynthesis and sugar metabolism. The 6938 DEGs among the materials included 1044 unique DEGs, and a total of nine statistically significant clusters were identified, which were mainly annotated in pathways related to photosynthesis, hormones and flavonoids. Three specific modules that were significantly related to the nutritional content of bean sprouts were identified via WGCNA. The connectivity and functional annotation of genes within the modules were calculated, and nine candidate genes were found, nine of which encoded transcription factors (*Glyma.16G071900* (WD40), *Glyma.17G172400* (bHLH), *Glyma.18G148000* (AP2) and *Glyma.01G003000* (MYB)). Conclusions: These research results provide a theoretical basis for an in-depth understanding of the molecular mechanisms of soybean sprout development and nutritional components and provide new genetic resources for the study of nutritional components in soybean sprouts.

## 1. Introduction

Soybean (*Glycine max*) is an annual herbaceous plant of the Leguminosae family. The life cycle of the soybean is usually one year, and it is a fast-growing plant. The growth period of soybean plants is short, and it usually takes 3–5 months from sowing to maturity, after which it blooms and bears fruit. After the fruit matures, it releases seeds and completes a life cycle. Soybeans live mainly in subtropical and tropical regions, prefer warm and humid climates, and have high temperature and light requirements. The suitable growth temperature is generally between 15 °C and 30 °C, and the optimum growth temperature is approximately 25–28 °C. Soybeans can generally grow in sufficient sunlight and well-drained soil. The antinutritional components of soybean seeds are problematic, which makes the human body inefficient in utilizing nutrients when these seeds are consumed [1]. After soybean seeds are soaked in water to germinate, their biochemical activities become active during the germination process, and the chemical substances of the nutrients are changed, making them more easily absorbed by the human body [2]. Like traditional vegetables, bean sprouts are loved by people because of their good taste, lack of pollution, high nutritional content and easy absorption by the human body [3]. Soybean sprouts are an important source of plant protein. They contain a variety of amino acids and can provide the protein needed by the human body. They are rich in vitamins such as vitamin C, which can enhance the body’s immunity and prevent various diseases. They are rich in minerals such as calcium, iron and zinc, which help promote bone health, blood circulation and cell metabolism. Soybean sprouts are rich in dietary fiber, which helps promote intestinal peristalsis and prevent constipation and intestinal diseases. Research has shown that the protein content and structure of soybean kernels significantly change after germination [4]. For example, experiments by Lee et al. demonstrated that the total protein content of germinated soybeans increased by approximately 21% compared with that of untreated soybean kernels [5]. Soybean seeds contain little or no vitamin C, and research has shown that 5 days after soybean germination, the vitamin C content in the buds reaches 20 mg/100 g [6]. There are great differences in the vitamin C contents of different varieties of soybeans within the same cultivation time. In recent years, changes in the fat content and composition of bean sprouts have attracted much attention from scholars [7]. Dhakal et al. reported that although the contents of unsaturated fatty acids such as linoleic acid and linolenic acid decreased during soybean germination, this trend was not obvious [8]. Some studies have also reported that the total fat content of soybeans decreases by 32.28% after 8 days of germination, the ratio of stearic acid to oleic acid gradually decreases with germination time, and the ratio of linolenic acid to linoleic acid gradually increases; however, the increasing trend of linoleic acid content is not obvious [9].

RNA sequencing (RNA-seq) is a method for detecting whole-genome gene expression in a high-throughput quantitative manner [10,11]. RNA-seq technology has been used for whole-genome transcriptome profiling of different crop species and has been widely used in studies of plant development and response to adverse stress [12]. The correlation of transcriptome dynamics under mild and severe drought stress in wild and cultivated chickpeas revealed metabolic pathways (e.g., phenylalanine metabolism) and biological processes (e.g., stomatal development) that are differentially regulated between genotypes, potentially impacting drought tolerance [13]. A rice regulatory network was constructed via transcriptome data from different tissues throughout the life cycle of the indica rice varieties Zhenshan 97 and Minghui 63; this network uses known transcription factors as bait to screen new regulatory networks for lignin metabolism, and an unbiased network based on tissue specificity was constructed to identify new regulators of glycerophospholipid metabolism [14]. A total of 1298 publicly published soybean transcriptome data points revealed that among the 56,044 annotated genes in the soybean genome, 52,737 genes were expressed in at least one sample. Unsupervised clustering revealed that these samples can be clustered into three groups, including tissues from aboveground parts, underground parts, seeds and seed-related parts. A total of 452 genes presented comparable and constant expression levels, indicating that they function as housekeeping genes; 1349 genes presented strongly biased expression patterns in specific tissues, and 3256 transcripts presented new spliced forms [15]. Through 40 transcriptome datasets of cultivated and wild soybean seed development and gene co-expression network analysis, it was found that the gene expression of the three gene cluster module systems significantly increased in cultivated soybeans compared with that in wild soybeans. An analysis of gene co-expression networks combined with known QTL site information revealed that the gibberellin synthesis gene *GA20OX* and the transcription factor gene NFYA were positively correlated with seed weight and oil content [16]. By constructing a gene co-expression network of soybean seeds, the core gene *GmJAZ3* was identified. The overexpression of this gene in soybeans promoted increases in seed size and grain weight, reduced fatty acid content, and increased protein content. Transcriptome analysis revealed that *GmJAZ3* significantly inhibited the expression of multiple cytokinin oxidase genes, *GmCKX*. *JAZ3* underwent artificial selection during the domestication process from wild soybean to cultivated soybean. Its homologous genes in rice and *Arabidopsis thaliana* also have similar functions [17].

After years of research, genes related to the seed development and quality of various plants have been identified through omics data, and gene regulatory networks have been constructed [18,19]. However, research on genes and regulatory modules related to regulating the nutritional components of soybean sprouts is rare. Understanding the changes in nutrients during the process of bean sprout morphology after germination, as well as the candidate genes and regulatory pathways related to nutritional components, will enrich soybean germplasm resources and utilization value. Therefore, in this study, Dongnong 254 and Heze small beans were selected as test materials to analyze the changes in nutrient content and RNA-seq data during the development of bean sprout morphology after soybean germination. Cluster analysis, differential expression analysis, and Gene Ontology (GO) and Kyoto Encyclopedia of Genes and Genomes (KEGG) enrichment analyses were performed on the sequencing data. By constructing a weighted gene co-expression network, candidate genes and regulatory pathways related to the nutritional components of soybean sprouts were identified. This study provides a theoretical basis for an in-depth understanding of the molecular mechanisms underlying differences in the nutritional components of soybeans and provides new genetic resources.

## 2. Materials and Methods

### 2.1. Plant Materials

**Figure 1:**
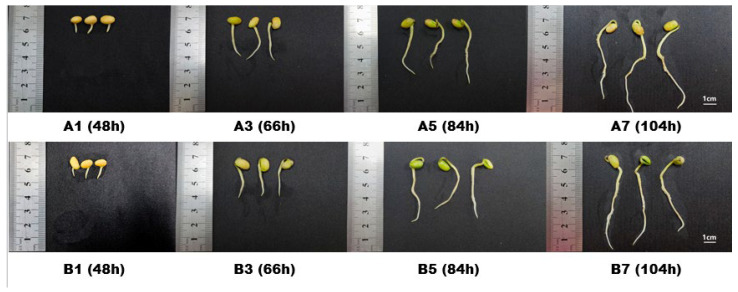
The bean sprout lengths of Dongnong 254 and Heze small beans were 1 cm, 3 cm, 5 cm and 7 cm, respectively. A is Dongnong 254, and B is Heze small bean.

This experiment used Dongnong 254 (100-seed weight of 7.6 ± 0.14 g, yellow seed coat, small round grains) and Heze small beans (100-seed weight of 8.15 ± 0.1 g, yellow seed coat, round small grains) as materials. Mature, plump and undamaged test soybean plants were selected from each test sample, rinsed three times with deionized water, and then soaked in 0.01% sodium hypochlorite solution for 15 min for disinfection. After disinfection, the samples were rinsed three times with deionized water. The rinsed samples were soaked in distilled water for 6 h (25 °C, dark conditions). The samples with good water absorption and undamaged skin were placed in a seedling tray covered with seedling paper in advance, the soybean seeds were covered with another layer of seedling paper, and the trays were placed in an environment of 25 °C and 85% humidity for cultivation. Deionized water was sprayed 1 to 3 times a day according to the actual conditions for germination treatment. After germination, samples were taken from bean sprouts that were 1 cm, 3 cm, 5 cm and 7 cm in length, quick-frozen in liquid nitrogen and stored at −80 °C for later use (Figure 1).

### 2.2. Determination of Nutritional Content

The sample was placed in a blast drying oven and dried at 40–50 °C. During this period, the sample was turned every hour until the weight of the sample did not change. After drying, the powder was ground with a pulverizer (XINNUO, Shanghai, China) for 60 s mesh sieve. The obtained crushed and sieved germinated soybean powder (referred to as bean sprout powder in subsequent experiments) was marked according to the four stages of bean sprout length (1 cm, 3 cm, 5 cm and 7 cm), placed in cryogenic tubes, and then frozen at −20 °C in a refrigerator for storage. The Kjeldahl method was used to determine the water-soluble protein content, the Soxhlet extraction method was used to determine the fat content, and the anthrone colorimetric method was used to determine the soluble sugar content, with three biological replicates each [20,21,22]. The determination of vitamin C requires the use of fresh samples; 3 samples were collected directly and stored in a −20 °C refrigerator, and the 2,6-dichloroindophenol titration method was used to determine the vitamin C content [23].

### 2.3. Transcriptome Library Construction and Sequencing

The extraction of RNA and transcriptome determination of the test samples were completed by Beijing Beiruihekang Biotechnology Co., Ltd. (Beijing, China). Total RNA was extracted using the RNAprep Pure Plant Kit (Tiangen, Beijing, China) according to the instructions provided by the manufacturer. The RNA concentration and purity were measured using a NanoDrop 2000 (Thermo Fisher Scientific, Wilmington, DE, USA). RNA integrity was assessed using the RNA Nano 6000 Assay Kit of the Agilent Bioanalyzer 2100 system (Agilent Technologies, Santa Clara, CA, USA). A total of 1 μg of RNA per sample was used as input material for the RNA sample preparations. The sequencing libraries were generated using the Hieff NGS Ultima Dual-mode mRNA Library Prep Kit for Illumina (Yeasen Biotechnology Co., Ltd., Shanghai, China) following the manufacturer’s recommendations, and index codes were added to attribute sequences to each sample. Briefly, mRNA was purified from total RNA using poly-T oligo-attached magnetic beads. The first strand of cDNA was synthesized, and the second strand of cDNA was subsequently synthesized. The remaining overhangs were converted into blunt ends via exonuclease/polymerase activities. After adenylation of the 3′ ends of the DNA fragments, NEBNext adaptors with hairpin loop structures were ligated to prepare for hybridization. The library fragments were purified with an AMPure XP system (Beckman Coulter, Beverly, MA, USA). Then, 3 μL of USER Enzyme (NEB, Ipswich, MA, USA) was incubated with size-selected, adapter-ligated cDNA at 37 °C for 15 min, followed by 5 min at 95 °C before PCR. Then, PCR was performed with Phusion High-Fidelity DNA polymerase, universal PCR primers and Index (X) Primer. Finally, the PCR products were purified (AMPure XP system), and library quality was assessed on an Agilent Bioanalyzer 2100 system. The libraries were sequenced on an Illumina NovaSeq platform (Illumina, San Diego, CA, USA) to generate 150 bp paired-end reads according to the manufacturer’s instructions.

### 2.4. RNA-Seq Analysis

The raw off-machine data were filtered and quality controlled via fastp. In this step, clean data (clean reads) were obtained by removing reads containing adapters, reads containing poly-N and low-quality reads from the raw data. Moreover, the Q20, Q30 and GC contents and sequence duplication levels of the clean data were calculated [24]. All downstream analyses were based on high-quality, clean data. The soybean genome (http://plants.ensembl.org/Glycine_max/Info/Index, accessed on 8 May 2024) was used as a reference, and TopHat2 was used for read comparison [25]. The Cuffquant and Cuffnorm components of Cufflinks software (vesion 2.2.1) were used to quantify the expression levels of transcripts through the position information of mapped reads on genes [26]. Gene expression was measured using the fragments per kilobase of transcript per million mapped reads (FPKM) method. Gene function was annotated via the following databases: Nr (NCBI nonredundant protein sequences), Pfam (Protein family), KOG/COG (Clusters of Orthologous Groups of proteins), Swiss-Prot (A manually annotated and reviewed protein sequence database), KO (KEGG Ortholog database) and GO (Gene Ontology).

### 2.5. Identification of DEGs

EdgeR was used to perform differential expression analysis to calculate the fold changes in gene expression between different samples based on the expression levels of genes [27]. An FDR ≦ 0.01 and an absolute value of log2-fold change ≧ 1 were used as the standards for screening differentially expressed genes (DEGs). Gene Ontology (GO) enrichment analysis of the DEGs was implemented via the clusterProfiler (vesion 4.16.0) package-based Wallenius noncentral hypergeometric distribution, which can adjust for gene length bias in DEGs. GO terms with corrected *p* values less than 0.05 were considered significantly enriched in DEGs. KEGG is a database resource for understanding high-level functions and utilities of biological systems, such as cells, organisms and ecosystems, from molecular-level information, especially large-scale molecular datasets generated by genome sequencing and other high-throughput experimental technologies (http://www.genome.jp/kegg/, accessed on 25 May 2024). We used the KOBAS database and clusterProfiler software (vesion 4.16.0) to test the statistical enrichment of DEGs in KEGG pathways [28,29].

### 2.6. Construction of the Coexpression Network

The R language WGCNA software (version 1.73) package was used to perform co-expression analysis on the gene expression profiles of DEGs through the dynamic branch-cutting method [30]. To ensure the distribution of the scale-free network, the weighting coefficient β should satisfy the correlation coefficient close to 0.8 and have a certain degree of gene connectivity. In this study, β = 14 was selected as the weighting coefficient. The automatic network construction function “Blockwise Modules” was used to construct the network. Multiple valid modules were obtained, and the number of genes contained in each module differed. Using minModuleSize = 30 and Merge Cut Height = 0.25 as the standards, modules with a similarity of 0.75 were merged. The correlation coefficient between the module’s characteristic vector module eigengene (ME) and different materials was calculated. The co-expression network was visualized via Cytoscape (version 3.10.1) software [31].

### 2.7. qRT–PCR

Total RNA was extracted using the RNAprep Pure Polysaccharide and Polyphenol Plant Total RNA Extraction Kit (Tiangen, China). The concentration of each RNA sample was determined using a NanoDrop 2000 spectrophotometer (Thermo Fisher Scientific, Waltham, MA, USA), and 1 μg of isolated RNA was subsequently used to obtain the first strand of cDNA via reverse transcription with a PrimeScript™ RT Kit with gDNA Eraser (Takara Bio Inc., Shiga, Japan). qRT–PCR analysis was performed using Roche LC480 equipment (Roche Diagnostics GmbH, Mannheim, Germany) and SYBR Green (Takara Bio, Inc.). A two-step PCR amplification procedure was used, with predenaturation at 95 °C for 30 s, followed by 40 cycles of denaturation at 95 °C for 5 s and annealing at 60 °C for 34 s. Amplification, dissolution and standard curves were automatically generated by Roche LC480 software (vesion 1.5). The results were analyzed relatively quantitatively using the 2^−ΔΔCt^ method. The internal reference gene was Act11, and each procedure involved three biological replicates [32]. All primers used in this study are listed in Table S1.

## 3. Results

### 3.1. Changes in the Nutrients of Different Soybean Sprouts

**Figure 2:**
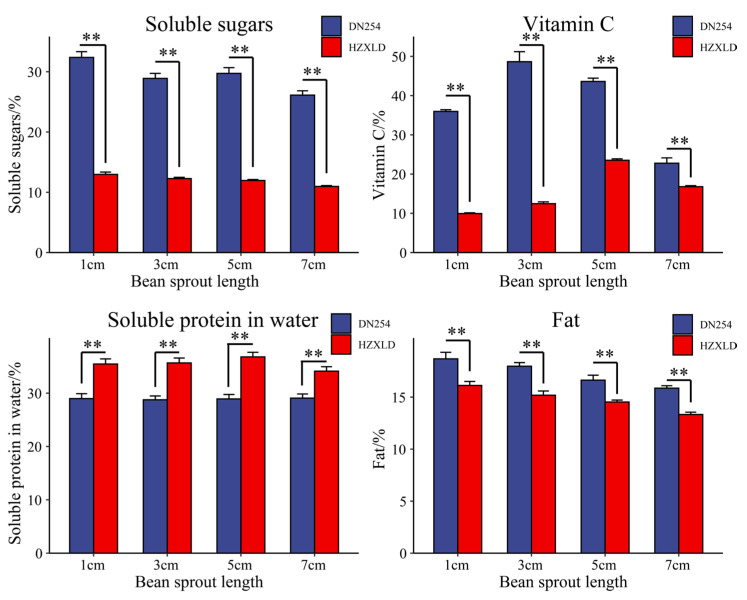
Nutrient contents of Dongnong 254 and Heze small bean sprouts at 1 cm, 3 cm, 5 cm and 7 cm in length. The error bars represent the average values ± SDs from three replicates (** *p* < 0.01).

By measuring the nutrients of Dongnong 254 and Heze small bean sprouts at 1 cm, 3 cm, 5 cm and 7 cm long, it was found that as the length of the bean sprouts increased, the soluble sugar and fat contents gradually decreased, the vitamin C content first increased and then decreased, and the soluble protein content in the water increased slightly. The soluble sugar, fat and vitamin C contents in Dongnong 254 beans were significantly greater than those in Heze small beans, and the soluble protein content in water was significantly lower than that of Heze small beans. To further identify the regulatory network and key genes involved in nutrient metabolism at different soybean sprout length stages, RNA-seq was performed on samples from plants with sprout lengths of 1 cm, 3 cm, 5 cm and 7 cm from these two materials (Figure 2).

### 3.2. Overall Analysis of the Transcriptome Sequencing Data

**Figure 3:**
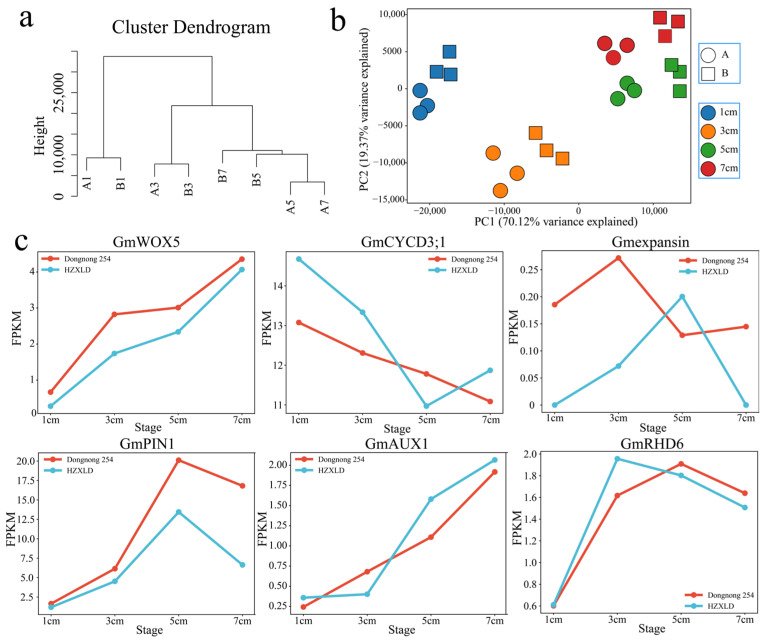
(**a**) Cluster dendrogram showing three different developmental stages; (**b**) PCA of 24 RNA-seq samples; (**c**) changes in the expression levels of root development marker genes (*GmWOX5*, *GmCYCD3;1*, *Gmexpansin*, *GmPIN1*, *GmAUX1* and *GmRHD6*). A is Dongnong 254, and B is Heze small bean.

After the raw data obtained in this study were filtered, 177.42 Gb of clean data were obtained. The amount of clean data from each sample was greater than 6.19 Gb, the GC content was greater than 44.36%, the Q20 and Q30 base percentages were greater than 96.24% and 90.18%, respectively, and the alignment rate with the reference genome was greater than 94.59% (Table S2). Ten genes were randomly selected for three independent qRT-PCRs, and the RNA-seq and qRT-PCR data were significantly correlated (R = 0.90, *p* < 0.01) (Figure S1). To analyze the transcriptome dynamics of soybean sprout length changes in depth, we performed PCA and hierarchical clustering on samples from two materials and four stages (Figure 3a,b). RNA-seq sample clustering and principal component analysis (PCA) revealed the greatest difference between samples at 3 cm and 5 cm, and these two periods resulted in the accumulation of the greatest amount of nutrients. Notably, the differences between the sample materials of Dongnong 254 and Heze small-grain bean sprouts with lengths of 5 cm and 7 cm are greater than those within the materials, which indicates that the main reason for the difference in nutrients between Dongnong 254 and Heze small-grain bean sprouts is the different expressions of a large number of transfers at lengths of 5 cm and 7 cm. The expression levels of soybean root development marker genes (*GmWOX5*, *GmCYCD3;1*, *GmEXPANSIN*, *GmPIN1*, *GmAUX1* and *GmRHD6*) were further analyzed (Figure 3c). All the root development marker genes presented similar expression trends in the two varieties, and the expression levels (FPKM) were also similar. These findings indicate that the RNA-seq data were reliable and reproducible and were suitable for further analysis.

### 3.3. Analysis of Differences Within Materials

**Figure 4:**
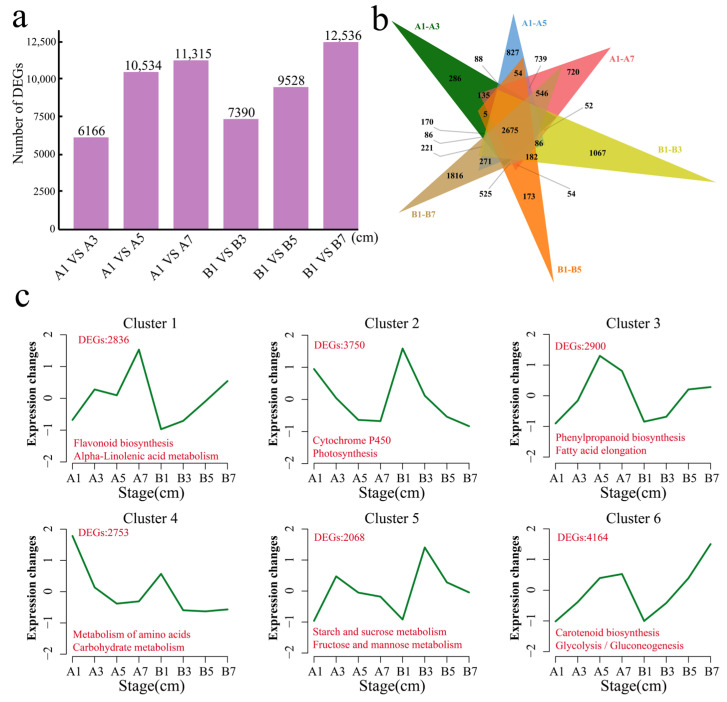
(**a**) Number of DEGs in different materials. (**b**) Venn diagram of DEGs within different materials. (**c**) Line chart of the expression patterns of DEGs within the material. A is Dongnong 254, and B is Heze small bean; the red numbers represent the number of DEGs contained in each cluster, and the colored text represents the significantly annotated KEGG pathways.

Difference analysis was performed on the same material at different development stages. In Dongnong 254, there were 6166 DEGs at 1 cm and 3 cm, including 286 unique DEGs; 10,534 DEGs at 1 cm and 5 cm, including 827 unique DEGs; and 10,534 DEGs at 1 cm and 7 cm, including 827 unique DEGs. There were 11,315 DEGs, including 720 unique DEGs (Figure 4a,b). In Heze small bean, there were 7390 DEGs at 1 cm and 3 cm, including 1067 unique DEGs; 9528 DEGs at 1 cm and 5 cm, including 173 unique DEGs; and 12,536 DEGs at 1 cm and 7 cm, including 1816 unique DEGs (Figure 4a,b). There were 2675 common DEGs between the two materials. Using the k-means clustering method, a total of six statistically significant clusters were identified for the 18,472 DEGs in the materials (Figure 4c). The expression level of cluster 1 in the two materials increased with increasing bean sprout length, and the expression level decreased slightly when the bean sprout length of Dongnong 254 was 5 cm. Cluster 1 contained 2836 genes that were significantly annotated in the flavonoid biosynthesis and *α*-linolenic acid metabolism pathways. The expression level of cluster 2 in the two materials decreased with increasing bean sprout length. Cluster 2 contained 3750 genes that were significantly annotated in the cytochrome P450 and photosynthesis pathways. In addition to the increase in expression level in Heze small beans, Cluster 3 also presented a decrease in expression level when the Dongnong 254 bean sprouts were 7 cm long. Cluster 3 contained 2900 genes that were significantly annotated in the phenylpropanoid biosynthesis and fatty acid elongation pathways. The expression level of cluster 4 in the two materials decreased when the bean sprouts were 3 cm long and then remained essentially unchanged as the bean sprouts grew. Cluster 4 contained 2753 genes that were significantly annotated in the metabolism of amino acids and carbohydrate metabolism pathways. The expression of cluster 5 in the two materials increased when the bean sprouts were 3 cm long and then decreased as the bean sprouts grew. Cluster 5 contained 2068 genes that were significantly annotated in the starch and sucrose metabolism and fructose and mannose metabolism pathways. The expression of cluster 6 in the two materials increased with increasing bean sprout length, and the increasing trend was more obvious in Heze small beans. Cluster 6 contained 4164 genes, which were significantly enriched in the garotenoid biosynthesis and glycolysis/gluconeogenesis pathways.

**Figure 5:**
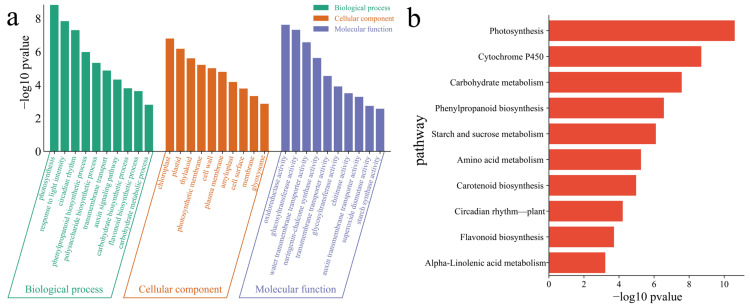
(**a**) GO enrichment analysis of DEGs within materials. (**b**) KEGG enrichment analysis of DEGs within the material.

To elucidate the functions of all 18,472 DEGs in the material, GO enrichment analysis was performed on the DEGs in the material (Figure 5a). The main annotations were related to the following biological processes: photosynthesis, response to light intensity, circadian rhythm, phenylpropanoid biosynthetic process, polysaccharide biosynthetic process, transmembrane transport, auxin signaling pathway, carbohydrate biosynthetic process, flavonoid biosynthetic process and carbohydrate metabolic process. The KEGG pathways associated with the DEGs between the materials included photosynthesis, cytochrome P450, carbohydrate metabolism, phenylpropanoid biosynthesis, starch and sucrose metabolism, amino acid metabolism, carotenoid biosynthesis, circadian rhythm, flavonoid biosynthesis and *α*-linolenic acid metabolism pathways (Figure 5b).

### 3.4. Analysis of Differences Between Materials

**Figure 6:**
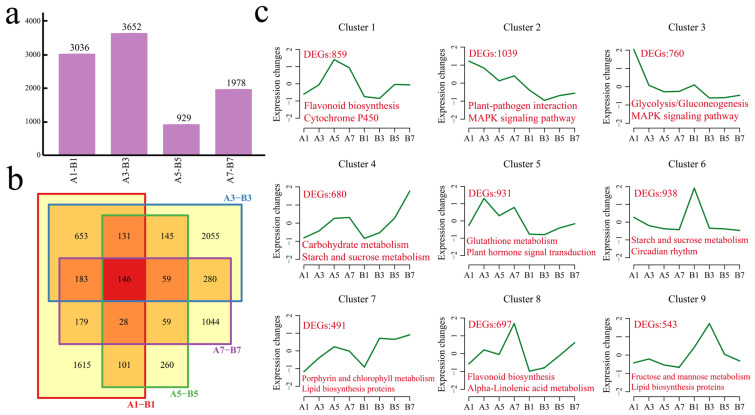
(**a**) Number of DEGs among different materials. (**b**) Venn diagram of DEGs between different materials. (**c**) Line chart of DEG expression patterns among materials. A is Dongnong 254, and B is Heze small bean; the red numbers represent the number of DEGs contained in each cluster, and the colored text represents the significantly annotated KEGG pathways.

Difference analysis was performed on the same developmental stages of different materials. At 1 cm, 3036 DEGs were detected in Dongnong 254 and Heze small beans, including 1615 unique DEGs. At 3 cm, Dongnong 254 and Heze small bean presented 3652 DEGs, including 2055 unique DEGs. At 5 cm, Dongnong 254 and Heze small bean presented 929 DEGs, including 260 unique DEGs. At 7 cm, there were 1978 DEGs in Dongnong 254 and Heze small bean, including 1044 unique DEGs (Figure 6a,b). There were 146 common DEGs between the two materials. Using the k-means clustering method, a total of nine statistically significant clusters were identified among the 6938 DEGs between materials (Figure 6c). The expression level of cluster 1 in the two materials increased as the bean sprout length increased, and the expression level decreased slightly when the bean sprout length of Dongnong 254 was 7 cm. Cluster 1 contained 859 genes that were significantly annotated in the flavonoid biosynthesis and cytochrome P450 pathways. The expression of Cluster 2 in Dongnong 254 decreased with increasing bean sprout length and reached a minimum at 5 cm. The expression in Heze small bean reached a minimum at 3 cm and then increased with increasing bean sprout length. Cluster 2 contained 1039 genes that were significantly annotated in the plant–pathogen interaction and MAPK signaling pathway. The expression level of cluster 3 in the two materials decreased with increasing bean sprout length. Cluster 3 contained 760 genes that were significantly enriched in the glycolysis/gluconeogenesis and MAPK signaling pathways. The expression level of cluster 4 in the two materials increased with increasing bean sprout length, and the increasing trend was more obvious in Heze small beans. Cluster 4 contained 680 genes that were significantly enriched in carbohydrate metabolism and starch and sucrose metabolism pathways. The expression level of cluster 5 increased in both materials, reaching a maximum value when the Dongnong 254 bean sprouts were 3 cm long. Cluster 5 contained 931 genes that were significantly enriched in the glutathione metabolism and plant hormone signal transduction pathways. The expression level of cluster 6 in Dongnong 254 was essentially unchanged, and the expression level was greatest in Heze small bean sprouts when the sprout length was 1 cm. Cluster 6 contained 938 genes that were significantly enriched in starch and sucrose metabolism and the circadian rhythm pathway. The expression level of cluster 7 in the two materials increased with increasing bean sprout length, and the increasing trend became more obvious when the bean sprout length of the Heze small bean was 3 cm. Cluster 7 contained 491 genes that were significantly annotated in the porphyrin and chlorophyll metabolism and lipid biosynthesis protein pathways. The expression level of cluster 8 in the two materials increased with increasing bean sprout length, peaking at a Dongnong 254 bean sprout length of 7 cm. Cluster 8 contained 697 genes that were significantly annotated in the flavonoid biosynthesis and *α*-linolenic acid metabolism pathways. The expression of cluster 9 in Dongnong 254 was essentially unchanged. It increased and then gradually decreased when the Heze small bean sprouts were 3 cm long. Cluster 9 contained 543 genes that were significantly annotated in the fructose and mannose metabolism and lipid biosynthesis protein pathways.

**Figure 7:**
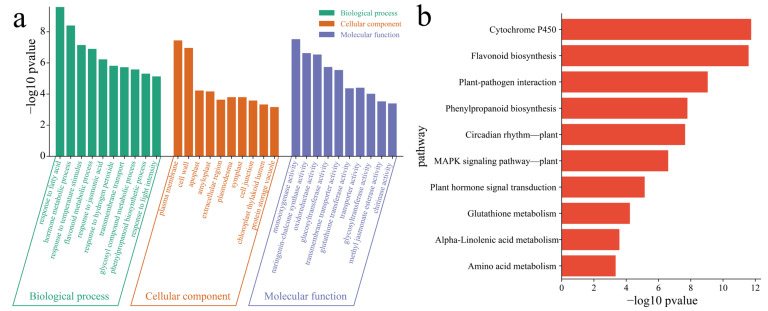
(**a**) GO enrichment analysis of DEGs between materials. (**b**) KEGG enrichment analysis of DEGs between materials. A is Dongnong 254, and B is Heze small bean.

To elucidate the functions of the 6938 DEGs between the materials, GO enrichment analysis was performed on the DEGs within the materials (Figure 7a). The main annotations were related to the following biological terms: response to fatty acids, hormone metabolic process, response to temperature stimulus, flavonoid metabolic process, response to jasmonic acid, response to hydrogen peroxide, transmembrane transport, glycosyl compound metabolic process, phenylpropanoid biosynthetic process and response to light intensity process. The KEGG pathways associated with the DEGs between the materials included cytochrome P450, flavonoid biosynthesis, plant–pathogen interaction, phenylpropanoid biosynthesis, circadian rhythm, the MAPK signaling pathway, plant hormone signal transduction, glutathione metabolism, *α*–linolenic acid metabolism and amino acid metabolism pathways (Figure 7b).

### 3.5. WGCNA

**Figure 8:**
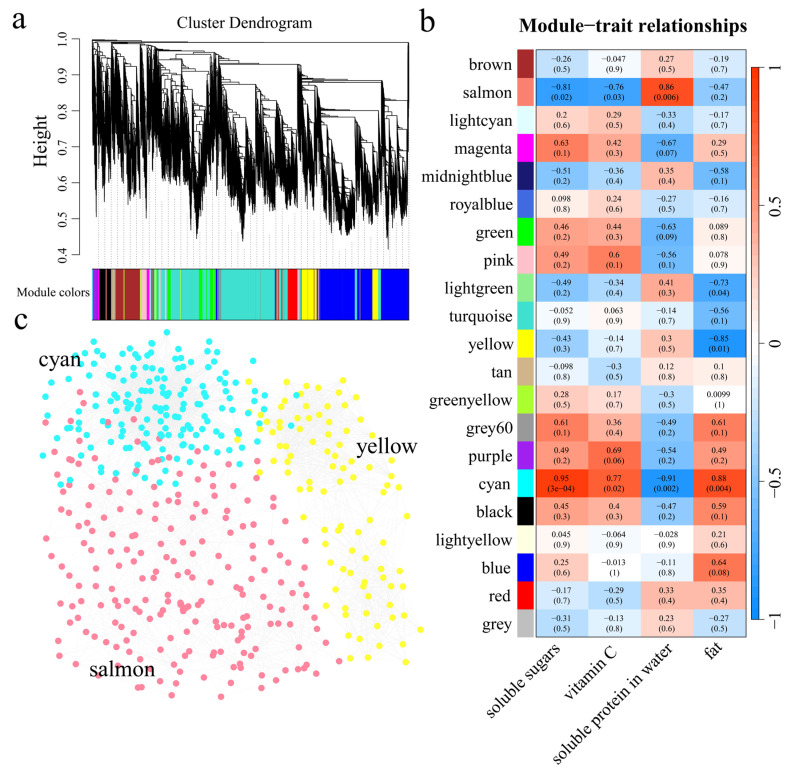
(**a**) Hierarchical clustering tree for gene coexpression network analysis. (**b**) Heatmap of the correlation and significance of modules associated with the nutritional components of bean sprouts. (**c**) Gene coexpression network of Salmon, yellow and cyan modules.

Weighted gene co-expression network analysis (WGCNA) was used to construct a co-expression network of 19,323 DEGs in soybean sprouts of different lengths. The dynamic shear tree method was used to merge modules with similar expression, and a total of 21 co-expression modules were obtained (Figure 8a). The salmon module was significantly positively correlated with the soluble protein content (r > 0.8, *p* < 0.05), the yellow module was negatively correlated with the fat content (r > −0.8, *p* < 0.05), the cyan module was significantly positively correlated with the soluble sugar and fat contents (r > 0.8, *p* < 0.05), and the cyan module was significantly negatively correlated with the soluble protein content (r > −0.8, *p* < 0.05) (Figure 8b). By calculating the kME (eigengene connectivity) value of each module gene, the gene with the highest absolute kME value was used as the hub gene of each module. Salmon, yellow and cyan were selected to construct the interaction network of genes and identify core genes (Figure 8c). Three core genes were identified for each module, resulting in a total of nine core genes. To further explain the relationships between the nine core genes and soybean sprout nutrients, they were annotated via the GO, KEGG, Pfam and nr databases (Table 1). Four of the genes encode transcription factors, namely, *Glyma.16G071900* (WD40), *Glyma.17G172400* (bHLH), *Glyma.18G148000* (AP2) and *Glyma.01G003000* (MYB). *Glyma.01G228700* encodes chalcone synthase (CHS), which is involved mainly in the biosynthesis of flavonoids. *Glyma.06G194900* and *Glyma.16G205200* encode the light-harvesting complex (LHC), which is involved mainly in the process of photosynthesis. *Glyma.19G046800* encodes ribulose bisphosphate carboxylase/oxygenase small subunit 2 (RBCS2), which is involved mainly in the process of carbon fixation in photosynthetic organisms. *Glyma.19G106800* encodes glyceraldehyde 3-phosphate dehydrogenase (GAPDH), which is involved mainly in the metabolism of glyoxylate and dicarboxylate. In summary, we screened nine candidate genes related to the nutrient content of soybean sprouts through transcriptome analysis. The results provide a theoretical basis for an in-depth understanding of the molecular mechanism underlying the nutrient content of soybean sprouts and provide new genetic resources for research on the nutrient content of soybean sprouts.

## 4. Discussion

After soybean seeds are soaked in water, many biochemical activities occur during the germination process, and enzyme catalysis causes changes in their nutritional content [33]. Proteins that cannot be directly absorbed and utilized by the human body due to the increase in protease activity during germination lead to changes in the protein nutritional components of soybean seeds [34]. Under the action of relevant enzymes, they are reorganized into soluble proteins, free amino acids and other substances that are highly absorbed by the human body. Polysaccharides are converted to monosaccharides that are easily absorbed by the human body. Changes in the structure of these nutrients are extremely beneficial for their absorption and utilization by the human body [35]. There is almost no vitamin C in soybean seeds, but the germination of soybean seeds greatly increases the vitamin C content. Vitamin C serves as an important “antioxidant” and “free radical scavenger” in food. It can play a very good protective role in maintaining human health and preventing diseases [36]. The American Institute of Cancer Prevention reported that the chlorophyll in bean sprouts can prevent rectal cancer. In addition, nitrophatases in soybean sprouts can supplement the enzymes that epilepsy patients lack and can play an auxiliary therapeutic role in the human body. Saponins, isoflavones and other bioactive substances present in bean sprouts have health-related effects on the human body [37]. This study selected Dongnong 254 and Heze small beans and reported that the soluble sugar and fat contents gradually decreased as the bean sprout length increased. This may be due to the need for fats to provide energy during seed germination and growth after germination. Therefore, it gradually decreases with increasing germination time. The vitamin C content first increased but then decreased. There is little or no vitamin C in mature soybean seeds. However, after germination, vitamin C reached its highest level and then gradually decreased over time. This is mainly due to internal participation in material metabolism after germination. The enzymes are produced or activated accordingly, and the increased metabolism of substances inside bean sprouts leads to increased enzyme activity for synthesizing vitamin C, which ultimately leads to an increase in the vitamin C content after germination. The content of soluble protein in the water increased slightly, mainly because during the entire germination and growth process, the nutritional structure of the protein changed, and the protein was used as an energy material for seed germination and growth in the early stage [38,39]. However, the type and proportion of subsequent amino acids and the protein content changed, so a wave-like change occurred, increasing the nutritional value of the soybean sprouts after germination [40]. Bean sprouts include different regions (root tips, hypocotyls and cotyledons). People eat bean sprouts as a whole as vegetables do, so we sampled bean sprouts as a whole. It is impossible to analyze the gene expression profiles and changes in nutrient contents in different parts of beans by studying bean sprouts as a whole. To gain a deeper understanding of the development process of bean sprouts, independent samples and analyses of different parts, such as the root tip, hypocotyl and cotyledon, are needed. This study can more comprehensively reveal the differences and interactions between different parts of bean sprouts and provide more detailed guidance for nutritional improvement and optimized production of bean sprouts.

Hormones play important roles in plant growth and development and can affect fruit quality and nutritional content. Hormones such as gibberellins can promote the synthesis and accumulation of protein in bean sprouts, thereby increasing the protein content of bean sprouts. Protein is an important nutrient in bean sprouts and is beneficial to human health [41]. Hormones can regulate the synthesis and decomposition of vitamins in bean sprouts, increasing their vitamin content. Vitamins have a certain impact on the body’s metabolism and immune function and can improve immunity [42]. Studies in tomatoes have shown that the accumulation of vitamin C in fruits is antagonistically regulated by auxin and abscisin through mitogen-activated protein kinases, auxin response factors, and MYB transcription factors [43]. Photosynthetic products synthesized in plant leaves are an important source of sugar accumulation in fruits. They are mainly transported over long distances through the phloem and finally unloaded into the fruit [44]. Sugar metabolism plays an important regulatory role in plants and has an important impact on plant growth and development, energy supply, and stress resistance [45]. Through enrichment analysis of the DEGs, pathways related to hormones, glucose metabolism, and photosynthesis were found to be significantly enriched. These findings indicate that these pathways play a dominant role in the regulation of nutrients during bean sprout growth. As a key enzyme system involved in glucosinolate synthesis, the cytochrome P450 family has been widely studied in Brassicaceae plants, but mainly in *A. thaliana* [46,47]. The two subfamilies CYP79 and CYP83 are the main members of the cytochrome P450 enzymes involved in glucosinolate biosynthesis and metabolism, and are involved mainly in the core structure synthesis pathway [48]. CYP79F1, CYP79F2 and CYP83A1 control mainly the formation of aliphatic glucosinolates; CYP79B2, CYP79B3 and CYP83B1 control mainly the formation of indole glucosinolates; and CYP79A2 and CYP83B1 control mainly the formation of aromatic glucosinolates [49]. DEGs both between and within materials were also significantly enriched in the cytochrome P450 pathway.

Studies have shown that the increase in the oil content of yellow seeds in *A. thaliana* is caused not only by an increase in the proportion of seed embryos but also by an increase in the efficiency of fatty acid synthesis in the embryos [50]. Studies have shown that the *TT2* and *TT8* genes can directly or indirectly regulate the function of fatty acid synthesis genes, thereby improving the efficiency of fatty acid synthesis in embryos [50]. The TT polar shadow is the first step in regulating flavonoid synthesis. The presence of flavonoids cannot be detected in the tt4 mutant, which also causes the seeds of the tt4 mutant to be yellow. Studies have shown that *A. thaliana* TT family mutants present a high oil content [51]. Among them, the mechanism underlying the high oil content of the tt2 and tt8 mutants has been preliminarily elucidated. Recent studies have shown that TTG1 phosphorylation mediated by *SHAGGY-like kinases 11/12* (*SK11/12*) enhances the synthesis of fatty acids in the seed embryo while inhibiting the synthesis of mucus and flavonoids in the seed coat [52]. Flavonoids are synthesized by competing with fatty acids. The substrate in turn inhibits fatty acid synthesis. We also screened a gene encoding CHS (*Glyma.01G228700*) as a candidate gene for regulating the nutritional content of bean sprouts.

MYB transcription factors play important regulatory roles in the establishment of plant cell morphological patterns and in growth and development [53]. Studies have shown that MYB transcription factors are involved in regulating the establishment of glandular hair cell morphological patterns, including cotton fiber development [54]. *GmMYB73* overexpression increased the lipid content in the seeds and leaves of transgenic *A. thaliana* plants, as well as in seed length and thousand-grain weight, and in the seeds and leaves of transgenic lotus plants and hairy roots of transgenic soybean plants [55]. *GmMYB181* is expressed only in floral tissues, and overexpression of *GmMYB181* changes the morphology, fruit size and plant structure of *A. thaliana* floral organs, including outwardly curled sepals, smaller siliques, increased lateral branches and reduced plant height [56]. *CrMYB68* negatively regulates the expression of *β-carotene hydroxylase 2* (*CrBCH2*) and *9-cis-epoxycorus-enoid dioxygenase 5* (*CrNCED5*) in the exocarp of citrus (*Citrus reticulata*) and promotes the accumulation of α- and β-carotene in the fruit exocarp [57]. The overexpression of tomato *SlMYB75* activates ethylene signaling pathway genes and simultaneously activates the expression of lipoxygenase C (LOXC), amino acid decarboxylase (AADC2) and terpene synthase (TPS) in response to ethylene and ABA treatment [58]. It promotes the synthesis of aroma substances in the metabolic pathways of fatty acids, carotenoids and amino acids and increases the content of soluble solids, especially the contents of characteristic aldehydes and terpenes, in tomatoes by up to 10 times. The overexpression of *GmJAZ3* improved seed size and weight, reduced fatty acid content, and increased protein content. *GmJAZ3* directly interacts with the jasmonic acid signaling pathway transcription factor *GmMYC2a* and inhibits *GmMYC2a*-mediated transcriptional activation of *GmCKX3-4*. GmJAZ3 can also interact with the cytokinin signaling pathway transcription factor GmRR18a, inhibiting the transcriptional activation effect of GmRR18a on *GmMYC2a* as well as *GmCKX3-4* and collaboratively regulating soybean seed size [17]. We screened nine candidate genes, including four transcription factor genes (*Glyma.16G071900* (WD40), *Glyma.17G172400* (bHLH), *Glyma.18G148000* (AP2) and *Glyma.01G003000* (MYB), through WGCNA. In summary, our research provides a theoretical basis for an in-depth understanding of the molecular mechanism of nutrient content in soybean sprouts and provides new genetic resources for research on nutrient content in soybean sprouts.

## 5. Conclusions

In this study, 18,472 DEGs were identified within the materials, and 6938 DEGs were identified between materials through transcriptome sequencing of Dongnong 254 and Heze small-grain beans at different sprout lengths. Nine candidate genes related to the nutritional components of soybean sprouts were screened via WGCNA. The importance of pathways related to photosynthesis, hormones, and flavonoids in the regulation of the nutritional components of bean sprouts was emphasized. These research results provide a theoretical basis for a deeper understanding of the molecular mechanisms of soybean sprout development and nutritional components, and provide new genetic resources for the study of the nutrient content of soybean sprouts. In the later stage, it is still necessary to independently sample and analyze different parts, such as the root tip, hypocotyl and cotyledon. This study can more comprehensively reveal the differences and interactions between different parts of bean sprouts and provide more detailed guidance for nutritional improvement and optimized production of bean sprouts.

## Figures and Tables

**Table 1:** Functional annotation of candidate genes.

Gene ID	Gene Name	Function Annotation
*Glyma.01G003000*	MYB	Pentose phosphate pathway
*Glyma.01G228700*	CHS	Flavonoid biosynthesis pathways
*Glyma.06G194900*	LHC	Photosynthesis pathways
*Glyma.16G071900*	WD40	Growth regulation and development pathways
*Glyma.16G205200*	LHC	Photosynthesis pathways
*Glyma.17G172400*	bHLH	Circadian rhythm pathways
*Glyma.18G148000*	AP2	Regulates cotyledon and leaf development
*Glyma.19G046800*	RBCS2	Carbon fixation in photosynthetic organisms pathways
*Glyma.19G106800*	GAPDH	Glyoxylate and dicarboxylate metabolism pathways

## Data Availability

The RNA-seq data presented in the study are deposited in the NCBI repository under accession number PRJNA1173926.

## Supplementary Materials

The following supporting information can be downloaded at https://www.mdpi.com/article/10.3390/genes16060692/s1, Table S1: All primers used in this study; Table S2: Summary statistics for RNA-seq analysis of soybean; Figure S1: Correlation analysis of RNA-seq and qRT-PCR data.
