# Supplementary figures and images for: Identifying Candidate Genes Related to the Nutritional Components of Soybean (Glycine max) Sprouts Based on the Transcriptome and Co-Expression Network
Source: Genes (Basel). 2025 Jun 6;16(6):692. doi: 10.3390/genes16060692 (PMC12193213; doi:10.3390/genes16060692)

$R = 0.90, p < 0.01$

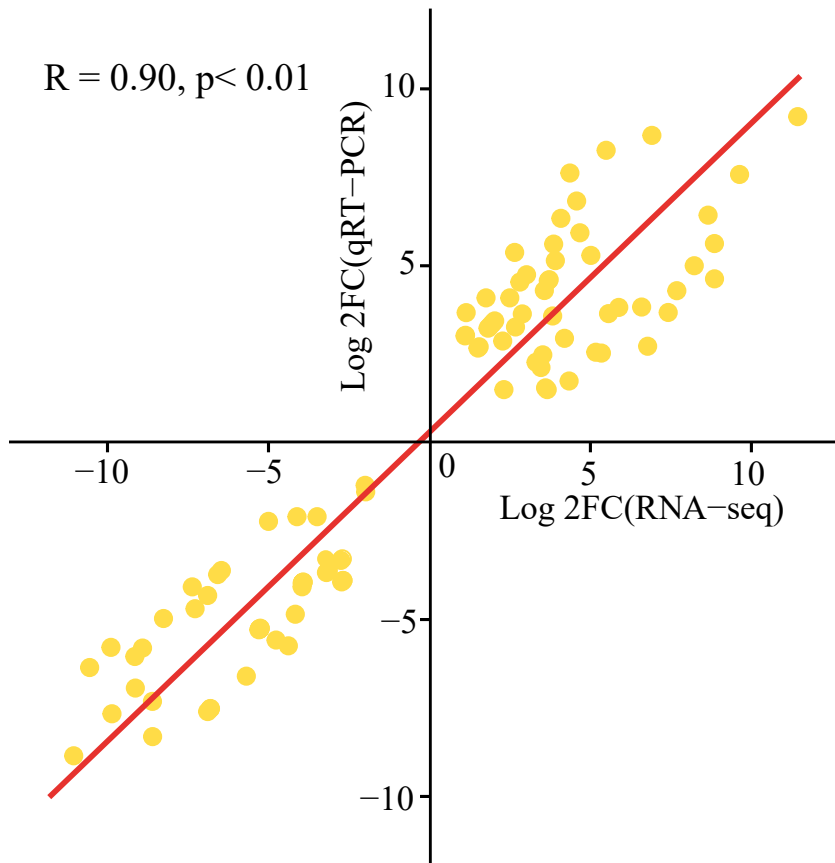

Supplement: Supplementary file 1 [file genes-16-00692-s001.zip › Fig S1.pdf]
